# Supplementary material for: Stacking Model‐Based Classifiers for Dealing With Multiple Sets of Noisy Labels
Source: Biom J. 2025 Mar 12;67(2):e70042. doi: 10.1002/bimj.70042 (PMC11898607; doi:10.1002/bimj.70042)
Supplement: Supplementary file 1 — Supporting Information [file BIMJ-67-e70042-s001.zip › density-based-ensemble-model-main/competitors_real_data/netlab-master/netlab-master/help/hinton.htm]

Netlab Reference Manual hinton


# hinton

## Purpose

Plot Hinton diagram for a weight matrix.

## Synopsis

```
hinton(w)
h = hinton(w)
```

## Description

`hinton(w)` takes a matrix `w`
and plots the Hinton diagram.

`h = hinton(net)` also returns the figure handle `h`
which can be used, for instance, to delete the
figure when it is no longer needed.

To print the figure correctly in black and white, you should call
`set(h, 'InvertHardCopy', 'off')` before printing.

## See Also

`demhint`, `hintmat`, `mlphint`

---

**Pages:**
Index


---

Copyright (c) Ian T Nabney (1996-9)
